# XPC Protects against Carcinogen-Induced Histologic Progression to Lung Squamous Cell Carcinoma by Reduced Basal Epithelial Cell Proliferation

**DOI:** 10.3390/cancers16081495

**Published:** 2024-04-13

**Authors:** Catherine R. Sears, Huaxin Zhou, Emily Hulsey, Bea A. Aidoo, George E. Sandusky, Nawar Al Nasrallah

**Affiliations:** 1 Pulmonary and Critical Care Section, Department of Medicine, Richard L. Roudebush Veterans Affairs Medical Center, Indianapolis, IN 46202, USA; 2 Division of Pulmonary, Critical Care, Sleep and Occupational Medicine, Indiana University School of Medicine, Indianapolis, IN 46202, USA; huaxzhou@iu.edu (H.Z.); nalnasra@iupui.edu (N.A.N.); 3 Department of Pathology and Laboratory Medicine, Indiana University School of Medicine, Indianapolis, IN 46202, USAgstandusk@iupui.edu (G.E.S.); 4 Department of Medicine, Indiana University School of Medicine, Indianapolis, IN 46202, USA; aaidoo@iu.edu

**Keywords:** DNA repair, non-small cell lung cancer, xeroderma pigmentosum, squamous cell, NTCU, dysplasia, tumor, progression, N-acetylcysteine, proliferation

## Abstract

**Simple Summary:**

Defects in DNA repair are considered critical to the development of genomic instability critical to cancer development. Here, we show the critical role of the DNA repair protein, Xeroderma Pigmentosum Group C (XPC), in protecting against the development and histologic progression of lung squamous cell carcinoma (LUSC). Using a murine NTCU carcinogen model, XPC-deficient mice are more likely to develop and have larger LUSCs compared with XPC wild-type mice. Using a time course, we show that mice deficient in XPC develop earlier progression of pre-cancerous histologic changes from dysplasia to LUSC. LUSC development in XPC-deficient mice originates in basal epithelial airway cells and is characterized by increased proliferation. Overall, this suggests that XPC plays a protective role in the development and progression of early LUSC, further characterization of which may lead to earlier detection and targets to improve survival.

**Abstract:**

Lung squamous cell carcinoma (LUSC) is the second leading cause of lung cancer. Although characterized by high DNA mutational burdens and genomic complexity, the role of DNA repair in LUSC development is poorly understood. We sought to better understand the role of the DNA repair protein Xeroderma Pigmentosum Group C (XPC) in LUSC development. XPC knock-out (KO), heterozygous, and wild-type (WT) mice were exposed topically to N-nitroso-tris-chloroethylurea (NTCU), and lungs were evaluated for histology and pre-malignant progression in a blinded fashion at various time-points from 8–24 weeks. High-grade dysplasia and LUSC were increased in XPC KO compared with XPC WT NTCU mice (56% vs. 34%), associated with a higher mean LUSC lung involvement (*p* < 0.05). N-acetylcysteine pre-treatment decreased bronchoalveolar inflammation but did not prevent LUSC development. Proliferation, measured as %Ki67+ cells, increased with NTCU treatment, in high-grade dysplasia and LUSC, and in XPC deficiency (*p* < 0.01, ANOVA). Finally, pre-LUSC dysplasia developed earlier and progressed to higher histologic classification sooner in XPC KO compared with WT mice. Overall, this supports the protective role of XPC in squamous dysplasia progression to LUSC. Mouse models of early LUSC development are limited; this may provide a valuable model to study mechanisms of LUSC development and progression.

## 1. Introduction

Lung cancer is common, with approximately one of every seventeen Americans expected to develop lung cancer in their lifetime [1]. Despite improvements in survival rates, lung cancer remains the leading cause of cancer death in the United States and worldwide, with 21% of all cancer deaths attributed to lung cancer [2]. This high mortality is largely attributed to the diagnosis of more than half of lung cancers at a late metastatic stage when 5-year survival is only 9% compared with 65% when diagnosed at a localized stage [3]. Lung squamous cell carcinoma (LUSC) is the second most common cause of lung cancer, accounting for approximately 30% of non-small cell lung cancers (NSCLCs) [4]. Diagnosing lung cancer early leads to improved survival, with early detection through lung cancer screening associated with a mortality reduction of 20–39% [5,6,7]. However, these gains are largely attributed to earlier diagnosis and improved therapies targeted at a specific histologic subset of non-small cell lung cancer (NSCLC), lung adenocarcinoma. The mortality benefit of lung screening in those with the second most common NSCLC, lung squamous cell carcinoma (LUSC), is less clear and likely reflects biological differences in NSCLC development and progression [8]. Additionally, improved biological, molecular, and immunophenotypic characterization is responsible for less toxic and more effective targeted therapies for a subset of patients with NSCLC. However, these have largely benefitted patients with the adenocarcinoma histologic subtype, with few LUSC benefitting from these targeted therapies [9]. Thus, enhancing our understanding of the early molecular changes in LUSC could potentially improve treatment strategies and patient outcomes. Genomic instability leading to the accumulation of mutations and genomic alterations is a hallmark of cancer and has been associated with impaired DNA repair processes [10]. LUSC development is strongly associated with exposure to carcinogens in tobacco smoke and is among the cancers with the highest levels of genomic alterations [10,11,12]. This suggests that impaired DNA repair may fundamentally impact LUSC development and progression. We and others have shown a protective role of the DNA repair protein xeroderma pigmentosum group C (XPC) in lung adenocarcinoma development, likely through alterations in both global genomic-nucleotide excision repair (GG-NER) and repair of oxidative DNA damage through base excision repair (BER) [13,14]. Mice deficient in XPC also develop an increased number of lung cancers with exposure to carcinogenic agents, including cigarette smoke [13,15,16]. We hypothesized that XPC would protect against lung squamous cell carcinoma development and progression and tested this using a mouse model of carcinogen-induced lung squamous cell carcinoma.

## 2. Materials and Methods

### 2.1. Chemicals

All chemicals, compounds, and reagents were provided by Thermo-Fisher Scientific (Waltham, MA, USA) unless otherwise noted. N-nitro-tris-chloroethylurea (NTCU) was obtained from Toronto Research Chemicals (North York, ON, Canada).

### 2.2. Animal Model

Mice used for these experiments were maintained in accordance with institutional guidelines and a lab animal protocol approved by the Indiana University School of Medicine Institutional Biosafety Committee (IBC, IN-972) and Institutional Animal Care and Use Committee (IACUC, #21132 and #21018). Male and female mice heterozygous for XPC in a mixed C57Bl/6;129 background were originally purchased from Jackson Laboratories, bred, back-crossed more than 10 generations, and genotyped prior to use as previously published [13,17]. Littermate male and female mice, XPC wild-type (XPC +/+; WT), heterozygous (XPC +/−), and knock-out (XPC −/−; KO) were used for these experiments [13]. NTCU was dissolved in acetone to 40 mM concentration or as indicated and applied to the shaved skin of the mice as previously published [18] with modifications as detailed in the text for weight-based dosing (1 µL/g body weight). Treatments [NTCU or acetone (control)] were applied twice a week to the shaved skin for the indicated duration or until morbidity/death. Mice that died or were sacrificed earlier than 19.5 weeks are reported but were excluded from histologic analyses. Mouse NTCU exposure experiments were performed in duplicate or triplicate for each treatment condition. When indicated, N-acetylcysteine (NAC) was provided ad libitum in drinking water (pH 7.4) at a concentration of 40 mM, changed three times a week. Blood, bronchoalveolar lavage, and lung harvest were performed as previously described [13]. Weights were checked twice per week, and mice noted to have weight loss on two consecutive measurements had NTCU treatment held until their weight recovered. Mice were sacrificed for continued weight loss (>20%) or other signs of distress.

### 2.3. Mouse Harvest, Tissue Specimen Collection and Processing

Mouse necropsy was performed as previously published [13]. Following anesthesia with isoflurane, the circulatory system was flushed, and bronchoalveolar lavage was performed as previously described with minor changes [17]. Briefly, 2 mL iced saline was instilled and removed by gentle syringe suction in 1 mL aliquots through a tracheostomy. Cellular and acellular components were separated via centrifugation, cell count was determined using trypan blue exclusion, and 28,000 cells were affixed to a slide by cytospin. Cells were fixed and stained using a Hema3 Manual Staining System (Fisher), and the cellular differential was determined in a blinded fashion by counting 500 cells from each slide. Images were taken using a Nikon Eclipse 90i and captured with NS Elements (Nikon, Tokyo, Japan). In the histology study, lungs were fixed in 10% neutral buffered formalin at 4 °C for 24 h following tissue processing and then embedded in paraffin. Multiple four-micrometer sections were obtained through the lung parenchyma, and the lung and bronchial epithelium were stained by routine hematoxylin and eosin stain (H&E).

### 2.4. Slide Evaluation for Histology and Percent Lung Calculations

H&E-stained lung sections (3–5 per mouse, in duplicate) were imaged using an Aperio (Reston, VA, USA) ScanScope CS at 40× magnification. The lesions were classified and quantified by two independent, blinded observers using the following grading criteria: a. flat atypia—characterized by bronchial epithelium composed of a single cell layer with normal bronchial epithelial cells interspersed between atypical cells that showed enlarged and hyperchromatic nuclei; b. metaplasia—characterized by flattened or no epithelium covering the basement membrane, varying from partial circumference of the airway to some with full circumference of the bronchiole airway; c. low-grade dysplasia—stratified non-ciliated squamous epithelial layer with maturation in upper layers as indicated by horizontal nuclear orientation and decreased nuclear-to-cytoplasmic (N:C) ratios, with atypia confined to the lower one-third to one-half of the epithelium; d. high-grade dysplasia—stratified, non-ciliated squamous epithelial layer without maturation showing high N:C ratios throughout the epithelium, with nuclear pleomorphism, variably-sized nuclei, hyperchromasia, loss of polarity and atypia that involved the full thickness of the epithelium; and e. invasive squamous cell carcinoma—nests of cells similar to high-grade dysplasia, with squamous differentiation, and illustrating invasion through the bronchial basement membrane.

Quantification of the percent lung involvement was performed in tumors or, if only pre-malignant changes were noted, in three to five bronchioles per slide, and the results averaged for each mouse by histology. To capture only the luminal epithelium, a line was drawn around the outer edge of the basement membrane below the epithelium and the inner lumen of the bronchiole using the Aperio ScanScope software (v12.4.3.5008). The whole lung cross-section was then demarcated, and the area was calculated to serve as the denominator to determine the percent lung involvement by histologic classification (metaplasia, low-grade dysplasia, high-grade dysplasia, squamous cell carcinoma).

### 2.5. Immunofluorescence Microscopy

Four-micrometer sections from formalin-fixed, paraffin-embedded lung specimens were deparaffinized and rehydrated prior to fluorescence immunohistochemical microscopy. Sections were evaluated for cytokeratin 5/6 (CK5/6, clone D5/16B4, Millipore (Burlington, MA, USA) at 1:100) or Ki67 (Abcam, Cambridge, UK, #16667, 1:100) by primary antibody conjugation, secondary anti-mouse or anti-rabbit AlexaFluor 488 and 594, and nuclei were counterstained with DAPI. Images were obtained using a Nikon Eclipse 90i and captured with NS Elements (Nikon). For multi-color fluorescence microscopy, overlaid images saved as .jpeg are shown. Mice were deidentified, and histologic characterization was performed.

### 2.6. Immunohistochemistry

Immunohistochemistry (IHC) staining was performed as previously published [13] using an antibody against Ki67 (Abcam #16667, 1:100) and hematoxylin for nuclear counterstaining. Images were obtained using a Nikon Eclipse 90i and captured with NS Elements (Nikon). Mice were deidentified, and histologic characterization was performed. Where appropriate, a line was drawn around the outer edge of the basement membrane below the epithelium and the inner lumen of the bronchiole to ensure quantification only of cells in the luminal epithelium. Percent Ki67 was determined by dividing Ki67+ cells by total cells (nuclei) and scored for each airway in a blinded fashion.

### 2.7. Statistical Analysis

Statistical analysis and production of graphical data were performed using SigmaPlot v14.5, where *p*-values < 0.05 were considered significant. Averages between two groups were performed using the two-tailed Student’s *t*-test and a comparison of the median by Mann–Whitney Rank Sum testing. Statistics for the Kaplan–Meier survival curves of multiple groups were performed using the Gehan–Breslow Test. Two-way ANOVA was performed for multiple conditions, with the Holm–Sidak method for multiple pairwise comparisons. Proportional analyses were performed using the Chi-Square or Fisher’s Exact test when fewer than 5 events occurred in any condition.

## 3. Results

### 3.1. XPC Deficiency Leads to Advanced Histologic Grade of Lung Squamous Cell Carcinoma

**Figure 1:**
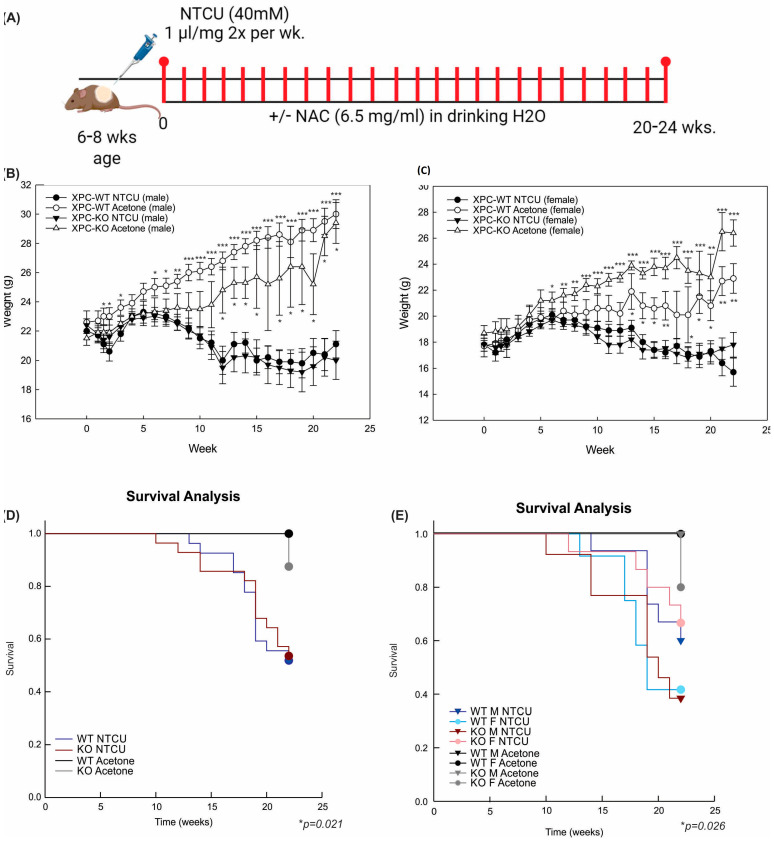
NTCU treatment causes weight loss and decreased survival, independent of XPC expression. (**A**) Schematic representation of N-nitroso-tris-chloroethylurea (NTCU) treatment. (**B**) Weights (mean +/− SEM) by week, treatment (NTCU or acetone control), and genotype (XPC WT, XPC KO) in all male and (**C**) female mice. * *p* < 0.05, ** *p* < 0.01, *** *p* < 0.001 using Student *t*-tests by treatment. (**D**) Kaplan–Meier survival analysis by XPC genotype and (**E**) XPC genotype and sex, showing a decrease in survival with NTCU treatment but not other variables. * *p* < 0.05 using Gehan–Breslow test.

We previously found that mice deficient in XPC develop squamous cell dysplasia when exposed to chronic cigarette smoke [13,17]. Based on these findings, we sought to determine the impact of XPC on lung LUSC development. Due to the low penetrance and long duration of cigarette smoking mouse models, we evaluated lung LUSC development by exposure to the carcinogen NTCU in XPC wild-type (WT) and knock-out (KO) mice [19]. Preliminary treatments were performed using published NTCU models, in which 40 mM NTCU was painted 25 µL on the shaved dorsum of both male and female mice twice a week [19]. We found that this was associated with unacceptably high mortality in female mice (both XPC WT and KO), which we attributed to lower body weight in female mice. Treatment with a lower dose, 25 µL of 8 mM NTCU twice a week, has been published to cause the development of pre-malignant squamous dysplasia in the mouse trachea and proximal bronchi [20]. Treatment with this low-dose NTCU was well-tolerated by mice and led to squamous atypia and tracheal dysplasia at 32 weeks. Consistent with these previous reports, no mice developed LUSC [20]. Based on the above experiments, we further refined the technique, and subsequent studies were performed by cutaneous painting of 40 mM NTCU at 1 µL/g body weight (Figure 1A).

**Figure 2:**
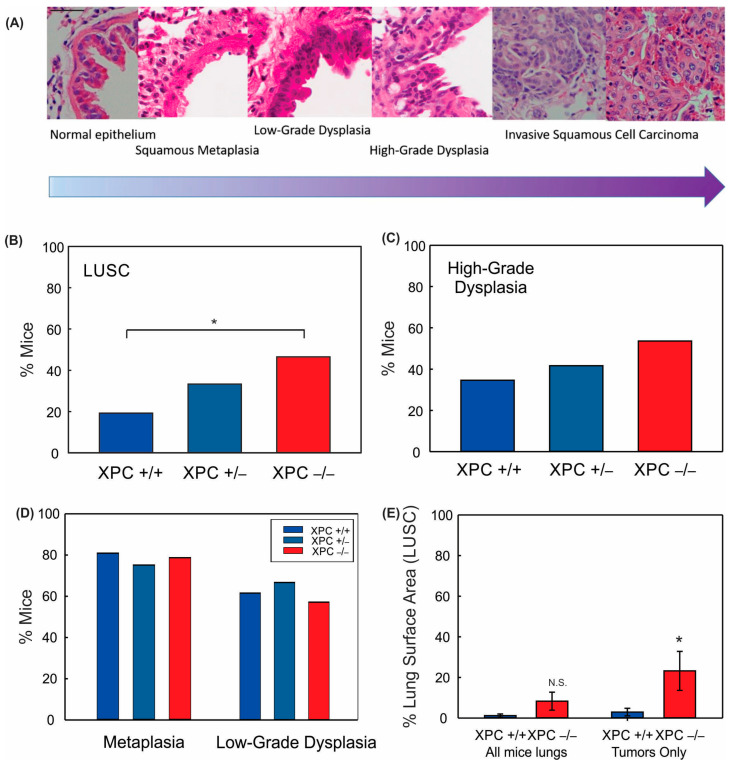
XPC protects against NTCU-induced lung squamous cell carcinoma (LUSC). (**A**) Characteristic hematoxylin and eosin (H&E) stained bronchial epithelium showing histologic progression characteristic of that seen in humans from normal bronchial epithelium to metaplasia to dysplasia to LUSC. 40x objective (bar 50 µm), images cropped. Percent mice with (**B**) LUSC, (**C**) high-grade dysplasia, (**D**) metaplasia, and low-grade dysplasia by XPC expression. (**E**) Percent LUSC lung surface area by XPC expression per mouse (all mice lungs) and in only those mice who developed LUSC (tumors only). * *p* < 0.05 using Chi-Squared or Student *t*-test. N.S. = not significant.

Treatment with 40 mM NTCU twice weekly at this dose was associated with persistent weight loss compared with treatment with acetone (Figure 1B,C). Survival was decreased in mice treated with NTCU compared with the acetone control (Table 1 and Figure 1D). Neither gender nor XPC expression was associated with differences in survival or weight loss in NTCU-treated mice (Table 1 and Figure 1C). Tracheal dysplasia was observed in both XPC WT and XPC KO mice, without overt malignancy observed in the trachea, consistent with the previously described NTCU models [20]. Both XPC WT and XPC KO mice treated with NTCU developed invasive LUSC within the lung (Figure 2A and Figure S1). Histologic advancement ranged from early pre-cancerous changes of atypia, metaplasia, low-grade and high-grade dysplasia, and ultimately, LUSC in both XPC KO and WT mice (Figure 2A). Mouse lungs were evaluated for the highest histologic grade at the time of necropsy or death. XPC deficiency was associated with a higher incidence of LUSC compared with XPC WT mice (46% vs. 19%; Figure 2B). XPC expression tended to protect against high-grade dysplasia in XPC KO, heterozygous, and WT mice, although this did not reach significance (Figure 2C). No differences in histologic findings of metaplasia or low-grade dysplasia were observed by XPC expression (Figure 2D). In those mice that developed tumors, LUSC was larger in XPC KO compared with WT mice, measured as percent lung surface area involvement (mean 23.215% vs. 2.94%, *p* < 0.05 using two-tailed Student *t*-test; median 12.353% vs. 1.532%, *p* = 0.017 using Mann–Whitney Rank Sum; Figure 2E). A minority of NTCU-treated mice (both XPC WT and KO) were incidentally noted to have granulomatous or lymphomatous lung lesions not associated with systemic disease by gross examination (Table 1 and Figure S2).

### 3.2. LUSC Development in XPC KO Mice Is Not Mitigated by N-Acetylcysteine or Antioxidant-Induced Changes to Alveolar Inflammation

In addition to its canonical role in the repair of large, strand-distorting adducts through NER, XPC is implicated in the repair of oxidative DNA lesions through the BER pathway [14,15,21]. Our laboratory previously discovered that preventative antioxidant treatment could mitigate lung adenocarcinoma development in XPC deficient mice, suggesting an important role of XPC regulation of BER in lung adenocarcinoma development, also suggested by others [13,14]. Therefore, we sought to investigate the role of inflammation and oxidative stress in LUSC development using XPC WT and KO mice.

Exposure to 40 mM NTCU led to inflammatory changes in the lung alveoli, as observed by increased bronchoalveolar lavage (BAL) cell count, which was significant in all mice compared with those painted with acetone control (Figure S3). BAL cell differential showed an increase in percent neutrophils and lymphocytes, with a subsequent relative decrease in the alveolar macrophage percentage (Figure S3).

**Figure 3:**
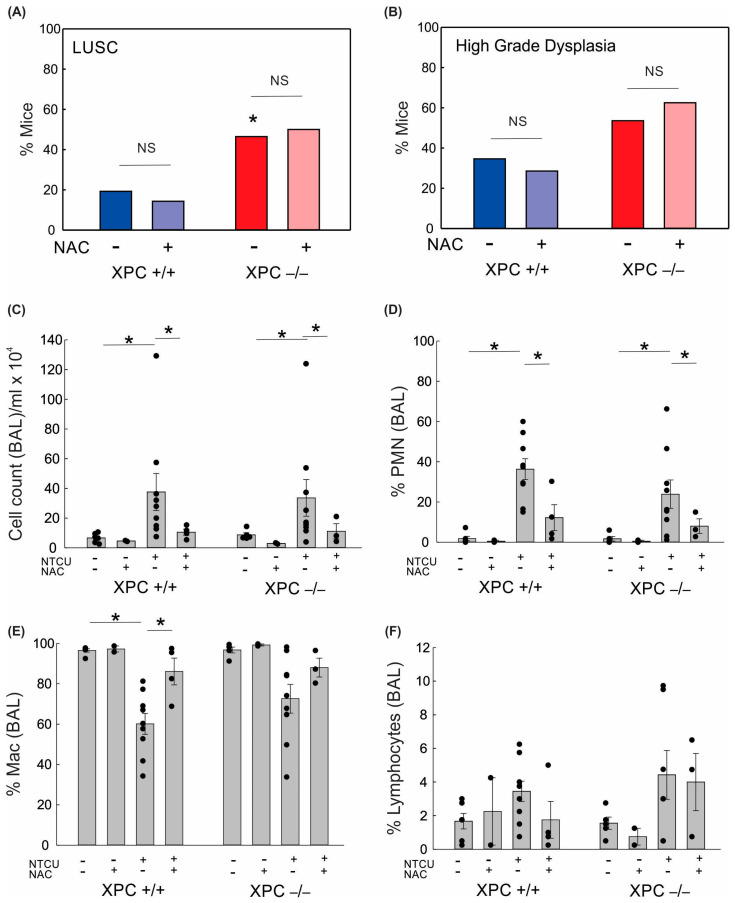
Impact of XPC and antioxidants on lung squamous cell carcinoma development and bronchoalveolar lavage. (**A**) Percent NTCU-exposed mice that developed LUSC by genotype and NAC treatment (**B**) Percent NTCU-exposed mice with high-grade dysplasia by genotype and NAC treatment. (* *p* < 0.05 using Fisher’s Exact test, compared with XPC +/+, n = 8 mice/group). (**C**) Total BAL cell counts per ml returned per mouse based on genotype, carcinogen exposure, and NAC treatment. (**D**) Percent polymorphonuclear cells in BAL. (**E**) Percent macrophages in BAL. (**F**) Percent lymphocytes in BAL. * *p* < 0.05 using two-way ANOVA.

Given the increased lung alveolar inflammation with NTCU treatment, we evaluated the role of oxidative stress on BAL immune cell counts and LUSC development. Treatment with the antioxidant N-acetylcysteine (NAC, 40 mM) in drinking water led to a reduction in alveolar lung inflammation as measured by cell count/mL BAL and percent polymorphonuclear cells in BAL (Figure 3). There was no difference in lung LUSC development or high-grade dysplasia development in NTCU-exposed mice treated with NAC (Figure 3). This suggests that the protective role of XPC in NTCU-induced LUSC is independent of oxidative stress.

### 3.3. Increased Histologic Progression of LUSC Development in XPC Deficiency Is Related to Basal Cell Proliferative Activity

**Figure 4:**
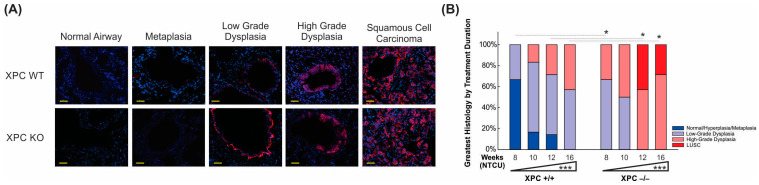
XPC deficiency is associated with earlier progression to pre-malignant and malignant histology. (**A**) Representative immunofluorescence microscopy pictures of CK 5/6 staining by histology, initially observed within the basal epithelial airway cells (low-grade dysplasia) and progressing to more uniform expression in high-grade dysplasia and LUSC. Scale bar = 100 µm. (**B**) Time course showing progression from normal/metaplastic to dysplasia and LUSC, with earlier, increased histologic progression in XPC KO compared with XPC WT mice. * *p* < 0.05, *** *p* < 0.001.

We proceeded to investigate the influence of XPC on LUSC development by examining CK 5/6 staining, a marker commonly used to identify LUSC [20]. The location and consistency of CK 5/6 varied based on the degree of dysplasia in the peripheral lung (Figure 4A). CK 5/6 staining was absent in airway epithelia histologically classified as normal or metaplastic/flat atypia. CK 5/6 staining was observed in airways with histologic changes consistent with low-grade dysplasia, where the distribution of CK 5/6 staining was observed in the basal epithelial cell layer. More diffuse expression of CK 5/6 to cells consisting of multiple layers of the bronchial epithelium was observed in high-grade dysplasia and LUSC. CK 5/6 staining did not differ by XPC gene expression when controlled for the histologic degree of bronchial dysplasia.

To further delineate the histologic progression of lung LUSC development in XPC WT and KO mice over time. Histologic characterization of the bronchial epithelium was performed by using H&E stain on FFPE sections from mice at the harvested 8, 10, 12, and 16 weeks after initiation of NTCU treatment. We found tracheal dysplasia in both XPC WT and KO mice, which was observed as early as 8 weeks after NTCU treatment. However, since LUSC primarily develops in the lungs and not the trachea, we focused on characterizing and quantifying airway histologic changes in lung sections in a blinded manner. Our analysis revealed that NTCU-exposed XPC KO mice exhibited a more advanced histologic grade of bronchial epithelium at 8, 12, and 16 weeks compared with NTCU-treated XPC WT mice (*p* < 0.001, Figure 4B and Supplemental Table S1), indicating an accelerated histologic progression of LUSC in XPC-deficient mice.

### 3.4. Airways of XPC KO Mice Show Increased Proliferation

**Figure 5:**
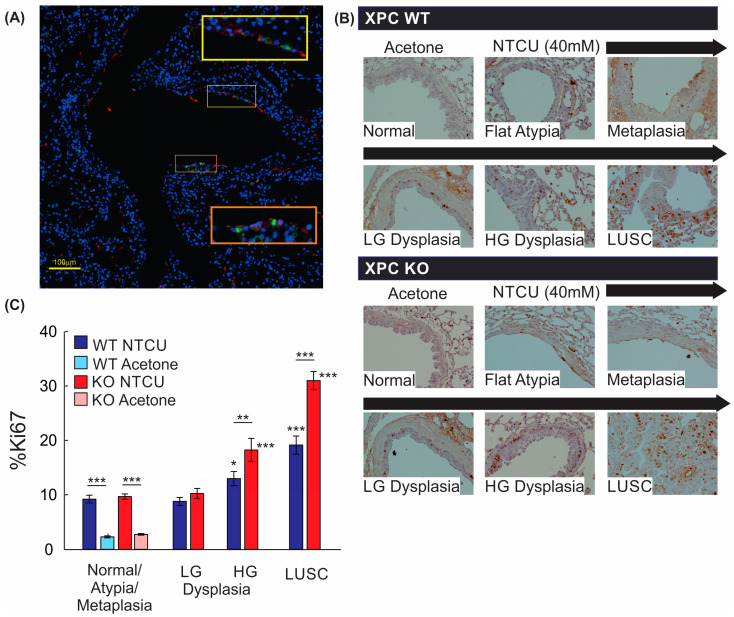
Proliferation by Ki67 staining is increased with NTCU, advancing histology, and in XPC KO. (**A**) Representative immunofluorescence microscopy image of a large airway showing co-localization of Ki67 and CK5/6 (orange and yellow squares with corresponding regions of interest blown up). 20× magnification. (**B**) Representative images of Ki67 staining by immunohistochemistry in XPC WT and KO bronchi by histology. Note the increase in Ki67+ cells in the basal epithelium of the airways. (**C**) Quantification of % Ki67+ cells by treatment (NTCU vs. acetone), XPC expression (XPC WT and KO), and histology. LG = low-grade dysplasia. HG = high-grade dysplasia. LUSC = lung squamous cell carcinoma. * *p* < 0.05, ** *p* < 0.01, *** *p* < 0.001 using two-way ANOVA.

We further evaluated the mechanistic impact of XPC on LUSC progression by evaluating cell proliferation, as measured by Ki67 staining. Within the bronchial airways, Ki67-expressing cells were primarily located within the basal layer of the epithelium, with Ki67+ stain co-localizing with CK5/6+ cells by immunofluorescence staining (Figure 5A,B). Next, we quantified Ki67+ cells within airway epithelial and LUSC cells. Treatment with NTCU alone increased the percentage of Ki67+ airway epithelial cells regardless of XPC expression, and Ki67 expression did not differ between airway epithelium classified as normal, flat atypia, metaplasia, or low-grade dysplasia by histologic appearance (Figure 5B). Compared with airways classified as normal, flat atypia, or metaplastic, more epithelial cells within regions of high-grade dysplasia and LUSC expressed Ki67+, with XPC KO mice showing an increase in %Ki67+ epithelial cells compared with XPC WT mice (Figure 5B). This suggests that the previously observed increase in high-grade dysplasia and LUSC in XPC KO mice correlates to an increase in proliferative activity.

## 4. Discussion

As highlighted in the introduction, lung squamous cell carcinoma (LUSC) poses a formidable challenge in oncology due to its aggressive nature and the limited treatment modalities available [8]. Understanding the molecular mechanisms underlying its development is crucial for the development of effective therapeutic strategies [22,23]. One such mechanism that has garnered attention is DNA repair, particularly the role of the Xeroderma Pigmentosum Group C (XPC) protein [15].

In this study, we used a mouse model to evaluate the role of the DNA repair protein, XPC, in LUSC development. We found that XPC expression protects against the development and progression of LUSC. Mice deficient in XPC are more likely to develop LUSC and progress to high-grade dysplasia earlier than mice expressing XPC. The mechanism by which accelerated LUSC progression occurs is related to the increased proliferation of basal cells within the airway epithelium.

Few mouse models are available to study the development of LUSC. Consistent with Ghosh et al. and others, we found early squamous dysplasia in the trachea, progressing to the proximal airways and, ultimately, the distal airways of the lung [18,20]. Further, our data supports the hypothesis that LUSC initiates from the proliferation of basal cells of the proximal bronchial epithelium, leading to histologic progression of squamous dysplasia from the proximal to distal bronchi and, ultimately, LUSC. Deficiencies in XPC appear to accelerate this process through this same mechanism by increasing basal epithelial cell proliferation and progression to earlier and more advanced squamous dysplasia and, ultimately, LUSC.

XPC deficiency was first discovered to play a critical role in the development of xeroderma pigmentosum, a condition characterized by early and aggressive melanomatous and non-melanomatous skin cancers, including squamous cell cancers of the skin [15]. Our findings complement the growing body of evidence suggesting a pivotal role of XPC in solid organ tumor development. Despite its implication in various malignancies, emerging evidence suggests a protective function against bladder and lung cancers [13,15]. Notably, XPC-deficient mice are predisposed to lung tumor development upon exposure to carcinogens and advancing age [13,24,25]. XPC is instrumental in facilitating global genomic nucleotide excision repair (GG-NER), tasked with rectifying bulky DNA lesions induced by various agents, such as UV light and tobacco smoke [15]. Furthermore, XPC contributes to base excision repair (BER), the primary mechanism responsible for rectifying minor DNA modifications caused by oxidative damage [15]. Previous studies, including our own, have implicated oxidative stress in the development of lung adenocarcinomas in XPC-deficient mice [13,14]. Moreover, XPC knockdown in keratinocytes was associated with elevated levels of reactive oxygen species through altered AKT1 and NOX1 pathways, resulting in squamous cell carcinomas of the skin [26]. While ad-lib access to NAC significantly decreased lung adenocarcinoma development in mice exposed to the carcinogen urethane in our previous study, NAC did not prevent LUSC development in either XPC proficient or deficient mice in the current model [13]. This suggests that nitrosamine-induced oxidative stress and the resultant DNA damage from the reactive oxygen species may not serve as the primary mechanism leading to NTCU-induced LUSC [27]. Rather, our findings support a mechanism by which XPC’s role in NER, BER, or both is protective against NTCU-alkylating DNA damage and resultant LUSC [28].

There are some limitations to our study. Mice used in this study express global knock-down of XPC, and therefore, the impact of XPC on specifically epithelial basal cells was not tested. Additionally, the tumor microenvironment is known to influence lung cancer development. Use of this global XPC knock-down mouse model does not allow for differential evaluation of XPC impact on other cells of the tumor microenvironment (e.g., stromal-mesenchymal and local tumor immune cells) and their potential role in LUSC development and proliferation. Although findings in mice may not completely recapitulate those in humans, the finding of characteristic histologic changes from squamous dysplasia to LUSC suggests LUSC tumor progression similar to that observed in humans. In our study, some mice developed granulomatous and lymphomatous lung lesions. C57Bl/6;129 mice are predisposed to lymphoma with age [29]. Although an increased incidence of lymphoma has been described in carcinogen models and DNA repair-deficient mouse models, the low incidence makes attribution to NTCU or XPC difficult [15,30,31]. We are not aware of any prior publication of granulomas with NTCU treatment; although concurrence of granulomatous inflammation with human cancers has long been described, its impact on lung cancer is largely unknown.

## 5. Conclusions

In conclusion, our study underscores the protective role of XPC in averting the onset of lung squamous cell carcinoma. Further research is warranted to characterize the critical genomic and transcriptomic alterations in the transition from dysplasia to LUSC. Additionally, further research should focus on the molecular mechanisms by which XPC impacts proliferation and its effect on DNA damage response, particularly in pre-malignant airway basal cells. Targeting XPC holds promise for future therapeutic interventions against LUSC.

## Figures and Tables

**Table 1:** Outcomes based on mouse genotype.

Genotype	Treatment	Deaths/Sac Prior to 19.5 Weeks (Total Mice)	Mice with LUSC/Total *	Mice with LUSC or High-Grade Dysplasia/Total *	Other Findings
XPC +/+	Acetone (control)	0 (13)	0/13	0/13	
XPC +/+	NTCU	11 (37) Death: 3 Sac: 8 ^Δ^	5/26 M: 3/17 F: 2/9	8/26 M: 5/17 F: 4/9	Granuloma (1), early lymphoma (2)
XPC +/−	Acetone (control)	0 (8)	0/8	0/8	
XPC +/−	NTCU	5 (17) Death: 5	4/12 M: 2/4 F: 2/8	5/12 M: 2/4 F: 3/8	Early lymphoma (2)
XPC −/−	Acetone (control)	0 (11)	0/11	0/11	
XPC −/−	NTCU	12 (40) Death: 5 Sac: 7 ^Δ^	13/28 M: 6/12 F: 7/16	16/28 M: 7/12 F: 9/16	Early lymphoma (1), Granulomas (3)

* At time of necropsy, excludes mice that died/sac < 19.5 weeks. ^Δ^ Sac= all sacrificed for severe/persistent cachexia other than two mice (XPC +/+, NTCU) that were sacrificed due to severe wounds/sores. LUSC = lung squamous cell carcinoma.

## Data Availability

Data are contained within the article and Supplementary Materials.

## Supplementary Materials

The following supporting information can be downloaded at https://www.mdpi.com/article/10.3390/cancers16081495/s1, Figure S1: Characteristic findings of the NTCU lung squamous cell (LUSC) model; Figure S2: Images characteristic of lymphomatous and granulomatous lung changes; Figure S3: Bronchoalveolar lavage (BAL) cellular component by NTCU treatment and XPC expression; Table S1: Progression of Histology by Weeks of NTCU Treatment.
