# Supplementary material for: XPC Protects against Carcinogen-Induced Histologic Progression to Lung Squamous Cell Carcinoma by Reduced Basal Epithelial Cell Proliferation
Source: Cancers (Basel). 2024 Apr 13;16(8):1495. doi: 10.3390/cancers16081495 (PMC11048415; doi:10.3390/cancers16081495)
Supplement: Supplementary file 1 [file cancers-16-01495-s001.zip › cancers-2939212-supplementary.pdf]

Supplemental Materials

Supplemental Tables and Figures

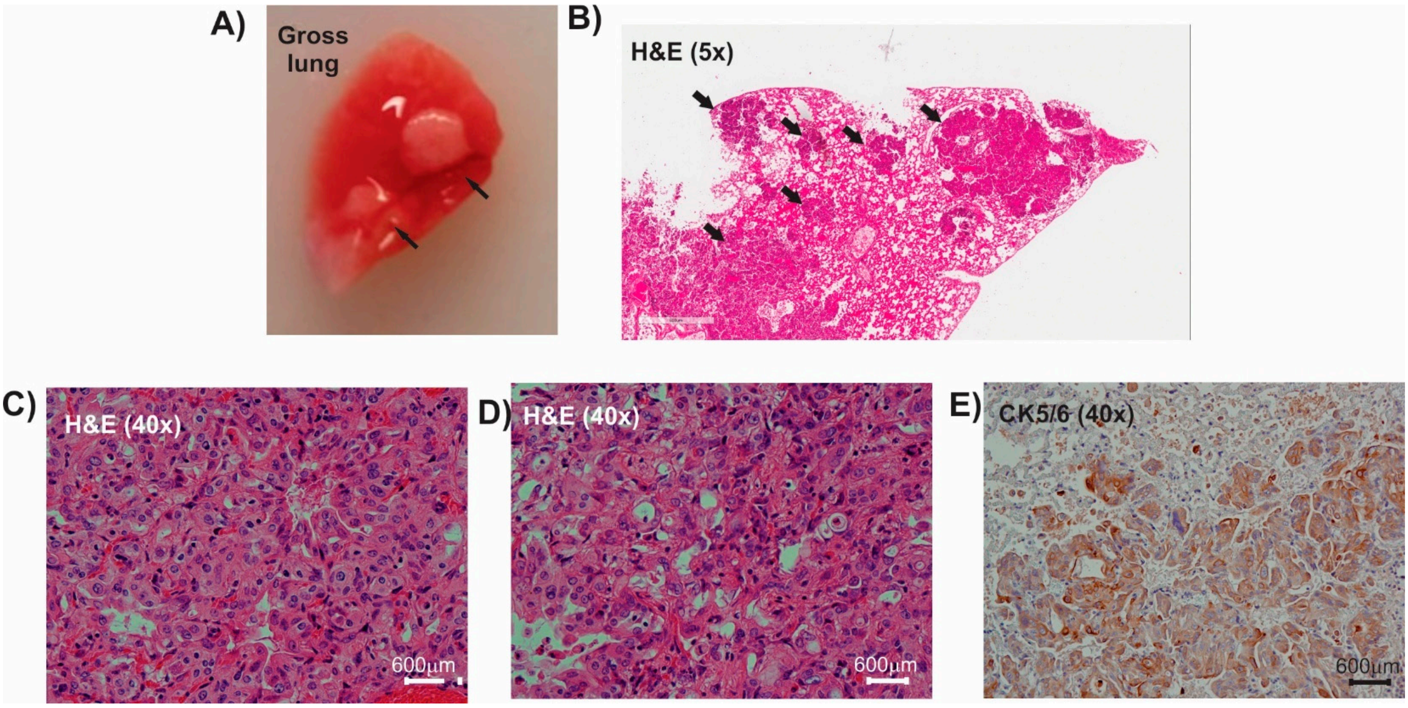

Figure S1: Characteristic findings of the NTCU lung squamous cell (LUSC) model A) Representative gross image of LUSC on a mouse lung surface. B) Characterization of LUSC by H&E stain at low (B) and high (C and D) magnifications. E) Characteristic positive CK 5/6 staining by immunohistochemistry. NTCU, N-nitroso-tris-chloroethylurea. LUSC, lung squamous cell carcinoma. CK5/6, cytokeratin 5/6. H&E, hematoxylin-eosin.

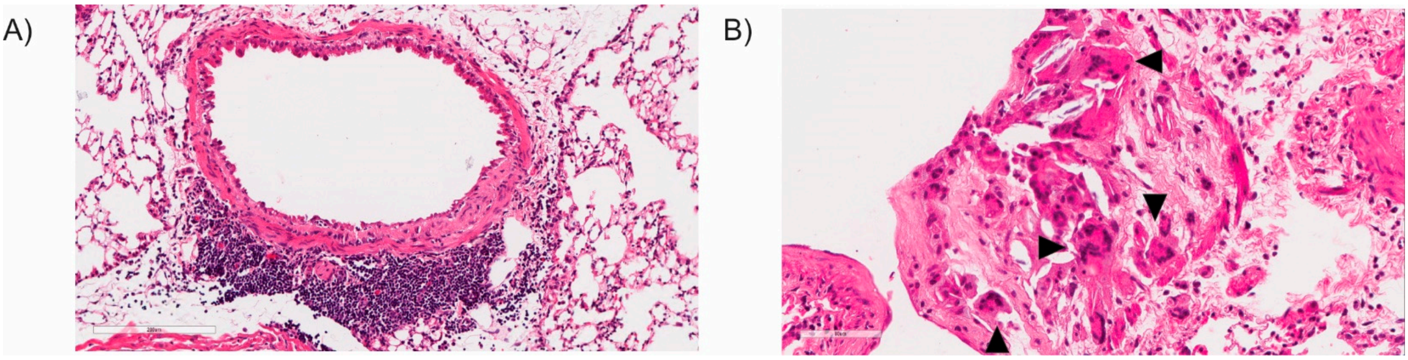

Figure S2: Images characteristic of lymphomatous and granulomatous lung changes. A) Lymphoid aggregates surrounding the airway. 20x magnification. B) Poorly formed granuloma with numerous giant cells (arrow heads). 30x magnification.

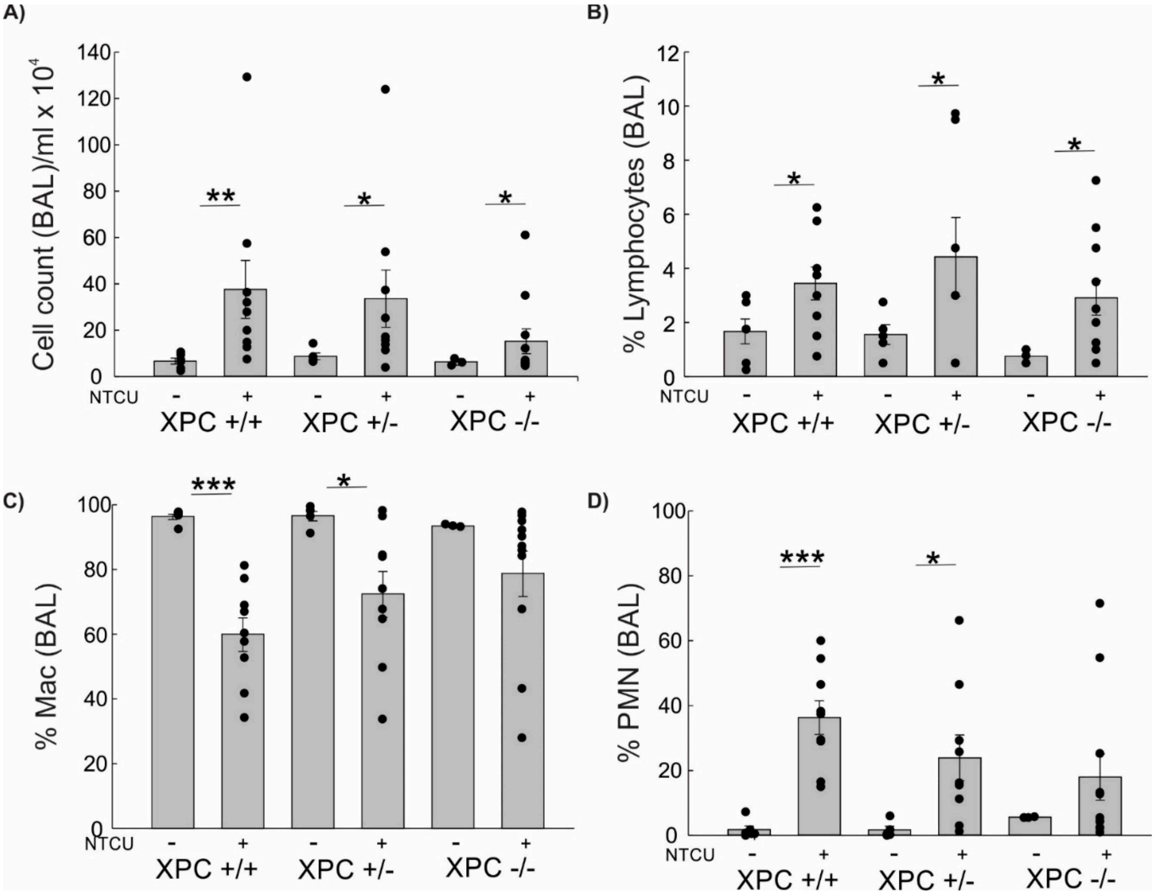

Figure S3: Bronchoalveolar lavage (BAL) cellular component by NTCU treatment and XPC expression A) Absolute cell count per ml BAL collected. B) Percent lymphocytes in BAL. C) % macrophages in BAL. D) % polymorphonuclear cells in BAL. *p<0.05, **p<0.01, ***p<0.001 by ANOVA with intergroup comparisons.

Supplemental Table. Progression of Histology by Weeks of NTCU Treatment.

Weeks (NTCU)	Highest Histologic Grade	Mice (total) by Genotype		p-value*
		XPC +/+	XPC -/-	
8 weeks				p=0.036
	Atypia/Metaplasia	4	0	
	Low-Grade Dysplasia	2	4	
	High-Grade Dysplasia	0	2	
	Squamous Cell Carcinoma	0	0	
10 weeks				p=0.343
	Atypia/Metaplasia	1	0	
	Low-Grade Dysplasia	4	3	
	High-Grade Dysplasia	1	3	
	Squamous Cell Carcinoma	0	0	
12 weeks				p=0.034
	Atypia/Metaplasia	1	0	
	Low-Grade Dysplasia	4	0	
	High-Grade Dysplasia	2	4	
	Squamous Cell Carcinoma	0	3	
16 weeks				p=0.039
	Atypia/Metaplasia	0	0	
	Low-Grade Dysplasia	4	0	
	High-Grade Dysplasia	3	5	
	Squamous Cell Carcinoma	0	2	

*Statistical difference in highest histologic grade by XPC genotype (p<0.001 overall by Fisher's Exact test, shown individually by genotype by weeks of NTCU treatment).
